# Gynecomastia after euthyroidism restoration in a patient with type 1 diabetes and Graves’ disease

**DOI:** 10.1002/ccr3.1565

**Published:** 2018-06-14

**Authors:** Valeria Calcaterra, Edoardo Clerici, Valeria Ceolin, Corrado Regalbuto, Daniela Larizza

**Affiliations:** ^1^ Pediatric Endocrinology Unit Department of Maternal and Children's Health Fondazione IRCCS Policlinico S. Matteo and University of Pavia Pavia Italy

**Keywords:** euthyroidism, Graves’ disease, gynecomastia, type 1 diabetes

## Abstract

In patients with autoimmune disease, gynecomastia should not be considered as 1 of the first signs of hyperthyroidism, rather it is a breast pathology that can be present even when euthyroidism restoration is achieved. It is unknown whether the autoimmune nature of thyroid disorders or simply the hyperthyroidism effects breast changes.

## 1 INTRODUCTION

This case report describes gynecomastia in a male patient with type 1 diabetes and Graves’ disease after thyroid function restoration had been achieved. Multifactorial mechanisms could be involved in the pathogenesis of breast enlargement and both thyroid function and thyroid autoimmunity may play a role.

Gynecomastia is defined as excess benign glandular growth of the breast tissue in males and it can be a physiologic or nonphysiologic condition.^1^, ^2^ Physiologic gynecomastia is common in newborns, adolescents, and older men; it occurs in up to 25% of patients and is self‐limited. No definitive cause is identifiable in another 25% of cases. In the remaining half of affected subjects, several conditions and drugs may induce proliferation of male breast tissue.^1^, ^2^, ^3^, ^4^

True gynecomastia (enlargement of the glandular tissue) is often caused by an imbalance between the stimulatory effect of estrogen and the inhibitory effect of androgens at the breast tissue level,^4^ as a consequence of different endocrine disorders.^1^, ^4^

An association between gynecomastia and thyroid dysfunction is uncommon, but has been well‐documented especially in hyperthyroidism.^1^ Thyroid hormones enhance the estrogen/androgen ratio by 2 main mechanisms: first, by direct stimulation of peripheral aromatase and thereby estrogen production, and secondly, by increasing hepatic SHBG synthesis.^5^ A relationship between autoimmune thyroid disease (ATD) and type 1 diabetes mellitus (T1DM) has also been described^6^, ^7^, ^8^; however, gynecomastia in diabetic patients with ATD has not been previously reported.

We describe a male patient with type 1 diabetes (T1D) who presented with gynaecomastia 3 months after starting methimazole treatment for Graves’ disease, when euthyroidism restoration had been achieved.

## 2 CASE REPORT

A 33‐year‐old subject, diagnosed with T1D at the age of 13 years, was seen in our out‐patient clinic in February 2017 for a routine follow‐up. At clinical evaluation, his general medical condition was good, moreover the patient had lost 3 kg body weight and his heart rate was 78 bpm. A physical examination confirmed the absence of goiter, hand tremors, exophthalmos, lid retraction, insomnia, palpitations, or other symptoms of hyperthyroidism. No abnormal breast changes were noted, no associated autoimmune diseases were detected at T1D diagnosis or during the routine diabetes follow‐up.

His laboratory investigations (Table 1) revealed high levels of free thyroxine (FT4) with a suppressed thyroid‐stimulating hormone (TSH) level (FT4 33.5 pmol/L, nv 10.3‐24.4; FT3 8.7 pmol/L, nv 2.7‐6.4; TSH 0.004 mUI/L, nv 0.04‐0.4), anti‐TSH receptor antibody positivity (2.7 U/L, nv < 1.75) and anti‐Tg‐A and anti‐TPO negativity. An ultrasound revealed a thyroid hypoechogenicity, without nodularity. Based on his symptoms, signs, and laboratory results, a diagnosis of Graves’ disease was made and 20 mg of methimazole was prescribed 3 times daily.

**Table 1:** Thyroid hormonal status, therapy, and gynecomastia evaluation during follow‐up

Parameters	Graves’ diagnosis	Follow‐up				
		2 months	3 months	5 months	7 months	9 months
TSH	0‐004 mcIU/L (nv 0.04‐0.4)	<0.01 mcIU/mL (nv 0.25‐ 5)	0.019 mcIU/L (nv 0.04‐0.4)	6.34 mcIU/mL (nv 0.25‐5.00)	3 mcIU/mL (nv 0.25‐5.00)	0.86 mcIU/mL (nv 0.25‐5.00)
FT4	33.5 pmol/L (nv 10.3‐24.4)	15.0 pmol/L (nv 9.0‐23.8)	10.0 pmol/L (nv 10.3‐24.4)	8.0 pmol/L (nv 9.0‐23.8)	12.1 pmol/L (nv 9.0‐23.8)	12.7 pmol/L (nv 9.0‐23.8)
FT3	8.7 pmol/L (nv 2.7‐6.4)	4.8 pmol/L (nv 3.1‐ 6.6)	4.7 pmol/L (nv 2.7‐6.4)	4.0 pmol/L (nv 3.1‐ 6.6)	4.2 pmol/L (nv 3.1‐ 6.6)	3.5 pmol/L (nv 3.1‐ 6.6)
Anti‐TSH receptor	2.7 IU/L (nv <1.75)			2.0 IU/L(nv <1.75)		neg
Methimazole prescription^a^	20 mg	15 mg	10 mg	5 mg	2.5 mg	Suspended
Gynecomastia	Absent	Absent	Present	Decreasing	Absent	Absent

a Dosage prescribed based on the results of hormone levels.

Two months later, the patient's thyroid hormone values improved (FT4 15.0 pmol/L, nv 9.0‐23.8; FT3 4.8 pmol/L, nv 3.1‐6.6); but the TSH level remained suppressed (TSH <0.01 mcU/mL, nv 0.25‐5). Thus, the methimazole dose was reduced to 15 mg 3 times daily (Table 1).

At the patient's 3 month follow‐up, he reported bilateral enlargement of his breasts (left more than right) that had started 15 days prior to the visit, without secretion from his nipples or signs of local inflammation.

The patient experienced limited swelling of the areola some days before developing visible gynecomastia. Ultrasonography confirmed gynecomastia. He denied a history of erectile dysfunction, decreased libido, breast manipulation, drug ingestion, or use of medications including oral contraceptive pills and other medications. A hormone panel showed a decreased FT4 level (10.0 pmol/L, nv 10.3‐24.4) and an initial TSH increase (0.019 mUI/L, nv 0.04‐0.4); therefore, the methimazole dose was further reduced to 10 mg twice daily (Table 1).

To investigate the causes of gynecomastia, plasma prolactin, plasma concentrations of sex hormone levels (including estradiol, testosteron, DHEA, DHEA sulfate, LH, and FSH hormones), sex hormone‐binding globulin, creatinine level, tumoral markers (carcinoembryonic antigen, alpha‐fetoprotein, cancer antigens 19‐9 and 125, beta‐ human chorionic gonadotropin), renal and hepatic parameters were measured and the results were normal, as shown in Table 2. Kidney and liver functions were preserved. Additionally, the patient was submitted to testicular, breast, and abdominal ultrasound, with normal results.

**Table 2:** Laboratory investigations at the time of the gynecomastia appearence

Parameters	Levels at evaluation	Normal values
Prolactin	11.9 ng/mL	2.5‐17
17‐beta‐estradiol	25.4 pg/mL	<56
Testosterone	397 ng/dl	300‐1080
Carcinoembryonic antigen	1.1 ng/mL	1.1
Alpha‐fetoprotein	4.5 IU/mL	4.5
Cancer antigens 19‐9	<1.2 IU/mL	<1.2
Cancer antigens 125	5.7 IU/mL	5.7
Beta‐ human chorionic gonadotropin	<2 mIU/mL	0‐5
Sex hormone‐binding globulin	60 nmol/L	13‐71
DHEA	7.2 nmol/L	5.55‐27.74
DHEA‐S	5.7 μmol/L	4.68‐11.70

At 5 months’ follow‐up, increased TSH values (6.34 mcU/mL, nv 0.25‐5.00) and decreased FT4 (FT4 8.0 pmol/L, nv 9.0‐23.8), were recorded. In parallel, an initial reduction of the gynecomastia was noted. The methimazole dose was again reduced to 5 mg daily (Table 1). At 7 months’ follow‐up (September 2017), his TSH and plasma concentrations of thyroid hormone had normalized (TSH 3 mcU/mL, nv 0.25‐5.00; FT4 12.1 pmol/L, nv 9.0‐23.8; FT3 4.2 pmol/L, nv 3.1‐6.6) and the gynaecomastia had disappeared. The methimazole dose was reduced to 2.5 mg daily (Table 1). At 9 months’ follow‐up, the patient was in great general condition, maintaining a euthyroid state; consequently methimazole therapy was stopped (Table 1).

The patient's thyroid hormonal status and therapies during follow‐up are summarized in Table 1.

## 3 DISCUSSION

Gynacomastia is the most common disorder of the male breast.^1^, ^4^ Interrelationships between thyroid alterations and gynecomastia have been reported and extend to both hypothyroidism and hyperthyroidism. Hypothyroid men have reduced testosterone secretion^9^ and often develop hyperprolactinemia. Whereas, hyperthyroidism is associated with increased peripheral conversion of androgens to estrogens and increased serum SHBG levels, which leads to decreased serum free testosterone.^10^, ^11^ Elevated serum levels of progesterone have also been reported in hyperthyroid men,^12^ although the biological significance of this observation and its relation to gynecomastia remain to be further evaluated.

Ten to 40% of men with thyrotoxicosis may develop gynecomastia that resolves with correction of the hyperthyroid state.^13^ To the best of our knowledge, this is the first report of a case of gynecomastia in a diabetic patient with Graves’ disease, in which the breast enlargement did not appear at the onset of hyperthyoridism, but rather after starting treatment with methimazole, when thyroid function restoration had already been achieved.

Gynecomastia is uncommon in type 1 diabetes. Hunfeld et al^14^, ^15^ reported 2 cases of diabetics suffering from gynecomastia with a marked inflammatory reaction, in which the lesion was interpreted as a diabetes‐induced autoimmune reaction of the breast. Devoto et al,^16^ described 2 patients with T1DM with chronic poor glycemic control and in a generally compromised nutritional state who developed gynecomastia after achieving glycemic restoration.

In our case, the gynecomastia was not associated with inflammatory signs or a compromised nutritional status and it appeared during a phase of euthyroidism restoration. Due to the normal levels of sexual hormones at the gynecomastia onset, a direct effect of thyroid hormone increasing the estrogen/androgen ratio was unlikely. Nevertheless, it is likely that there is no singular mechanism explaining the interaction between thyroid hormone and estrogen, but that there are multiple points of overlap in their signaling pathways, such as similar downstream signaling pathways and heteromeric nature of steroid hormone receptors,^17^ for this reason an imbalance between the effects of estrogen stimulation and androgen inhibition on the breast tissue during the phases of the thyroid disfunction should be considered.^18^ On the other hand, the gynecomastia improvement was also associated with decreases in the anti‐TSH receptor titer. In the literature, the association between breast diseases and thyroid autoantibodies has been reported,^19^, ^20^, ^21^ therefore, even though gynecomastia is described also in toxic multinodular goiter,^22^, ^23^ a causative role of thyroid autoantibodies should not be overlooked.^24^

Gynecomastia may also arise following the assumption of certain medications, or as a result of chronic kidney or liver diseases.^4^ Drugs that have been definitely associated with the onset of gynecomastia include spironolactone, cimetidine, ketoconazole, hGH, estrogens, hCG, anti‐androgens, GnRH analogs, and 5‐α reductase inhibitors. Medications probably associated with gynecomastia include risperidone, verapamil, nifedipine, omeprazole, alkylating agents, HIV medications, anabolic steroids, alcohol, and opioids.^25^ Men with chronic renal and liver failure also have raised estradiol and lowered testosterone concentrations which is sufficient for the development of gynaecomastia. Changes in circulating hormones are complex and affected by a combination of factors, including production, transport, and conjugation. In this report, drug assumption, chronic renal, and hepatic failure were excluded, so it seems reasonable to propose a relationship between the thyorid dysfunction and gynaecomastia in this patient. Because certain diseases of the breast are more closely associated with autoimmune thyroid phenomena (e.g. breast cancer),^19^, ^21^ whereas other diseases of the breast are associated with a hyperthyroid etiology (e.g. gynecomastia),^1^, ^2^, ^3^, ^13^ it remains unclear whether the autoimmune nature of thyroid disorders or simply the hyperthyroid state effects pathological breast changes.

In conclusion, this case report demonstrated that gynecomastia may appear during hyperthyrodism treatment in diabetic patients with Graves’ disease. Multifactorial mechanisms could be involved in the pathogenesis of breast enlargement and both thyroid function and autoantibodies may play a role.

## CONFLICT OF INTEREST

None declared.

## AUTHOR'S CONTRIBUTIONS

VC: management of the patient, literature review, drafted the article, critical revision of the article. EC, VCe and CR: drafted the article, literature review. DL: critical revision of the article. All of the authors read and approved the final manuscript.

## ETHICAL APPROVAL

The study was performed according to the Declaration of Helsinki and with the approval of the hospital's Institutional Review Board. The patient's parents involved in this study gave their informed consent authorizing use and disclosure of their protected health information.

## References

[1] Sansone A , Romanelli F , Sansone M , Lenzi A , Di Luigi L . Gynecomastia and hormones. Endocrine. 2017;55:37‐44. 27145756 10.1007/s12020-016-0975-9

[2] Fagerlund A , Lewin R , Rufolo G , Elander A , Santanelli di Pompeo F , Selvaggi G . Gynecomastia: a systematic review. J Plast Surg Hand Surg. 2015;49:311‐318. 26051284 10.3109/2000656X.2015.1053398

[3] Ordaz DL , Thompson JK . Gynecomastia and psychological functioning: a review of the literature. Body Image. 2015;15:141‐148. 26408934 10.1016/j.bodyim.2015.08.004

[4] Leung AKC , Leung AAC . Gynecomastia in infants, children, and adolescents. Recent Pat Endocr Metab Immune Drug Discov. 2017;10:127‐137. 28260521 10.2174/1872214811666170301124033

[5] Ismail AAA , Barth JH . Endocrinology of gynaecomastia. Ann Clin Biochem. 2001;38:596‐607. 11732643 10.1258/0004563011900993

[6] Shun CB , Donaghue KC , Phelan H , Twigg SM , Craig ME . Thyroid autoimmunity in Type 1 diabetes: systematic review and meta‐analysis. Diabet Med. 2014;31:126‐135. 24103027 10.1111/dme.12318

[7] Kahaly GJ , Hansen MP . Type 1 diabetes associated autoimmunity. Autoimmun Rev. 2015;15:644‐648. 10.1016/j.autrev.2016.02.017 26903475

[8] Nederstigt C , Corssmit EP , de Koning EJ , Dekkers OM . Incidence and prevalence of thyroid dysfunction in type 1 diabetes. J. Diabetes Complications. 2016;30:420‐425. 26868720 10.1016/j.jdiacomp.2015.12.027

[9] Meikle AW . The interrelationships between thyroid dysfunction and hypogonadism in men and boys. Thyroid. 2004;14:S17‐S25. 15142373 10.1089/105072504323024552

[10] Birzniece V . Doping in sport: effects, harm and misconceptions. Intern Med J. 2015;45:239‐248. 25369881 10.1111/imj.12629

[11] Dobs EA . Drug‐induced gynecomastia. Expert Opin Drug Saf. 2008;7:691‐702. 18983216 10.1517/14740330802442382

[12] Chentli F , Bekkaye I , Azzoug S . Feminizing adrenocortical tumors: literature review. Indian J Endocrinol Metab. 2015;19:332‐339. 25932386 10.4103/2230-8210.152764 PMC4366769

[13] Sanyal T , Dutta D , Shivprasad K , Ghosh S , Mukhopadhyay S , Chowdhury S . Gynaecomastia as the initial presentation of thyrotoxicosis. Indian J Endocrinol Metab. 2012;16:S352‐S353. 23565425 10.4103/2230-8210.104089 PMC3603073

[14] Hunfeld KP , Bässler R . Lymphocytic mastitis and fibrosis of the breast in long‐standing insulin‐dependent diabetics. A histopathologic study on diabetic mastopathy and report of ten cases. Gen Diagn Pathol. 1997;143:49‐58. 9269908

[15] Hunfeld KP , Bässler R , Kronsbein H . “Diabetic mastopathy” in the male breast–a special type of gynecomastia. A comparative study of lymphocytic mastitis and gynecomastia. Pathol Res Pract. 1997;193:197‐205. 9198105 10.1016/s0344-0338(97)80077-5

[16] Devoto CE , Madariaga AM , Aravena L , Lioi CX . Etiological study of gynecomastia: results of a prospective study and recommendations. Rev Med Chil. 2007;135:189‐197. 17406736 10.4067/s0034-98872007000200007

[17] Duarte‐Guterman P , Navarro‐Martín L , Trudeau VL . Mechanisms of crosstalk between endocrine systems: regulation of sex steroid hormone synthesis and action by thyroid hormones. Gen Comp Endocrinol. 2014;203:69‐85. 24685768 10.1016/j.ygcen.2014.03.015

[18] Harmeet Singh Narula MD , Harold E , Carlson MD . Gynecomastia. Endocrinol Metab Clin North Am. 2007;36:497‐519. 17543732 10.1016/j.ecl.2007.03.013

[19] Angelousi A , Diamanti‐Kandarakis E , Zapanti E , Nonni A , Ktenas E , Mantzou A , et al. Is there an association between thyroid function abnormalities and breast cancer? Arch Endocrinol Metab. 2017;61:54‐61. 28273204 10.1590/2359-3997000000191 PMC10522124

[20] Anil C , Guney T , Gursoy A . The prevalence of benign breast diseases in patients with nodular goiter and Hashimoto's thyroiditis. J Endocrinol Invest. 2015;38:971‐975. 25827711 10.1007/s40618-015-0269-8

[21] Prinzi N , Baldini E , Sorrenti S , De Vito C , Tuccilli C , Catania A , et al. Prevalence of breast cancer in thyroid diseases: results of a cross‐sectional study of 3,921 patients. Breast Cancer Res Treat. 2014;144:683‐688. 24604093 10.1007/s10549-014-2893-y

[22] Kapcala LP . Galactorrhea and thyrotoxicosis. Arch Intern Med. 1984;144:2349‐2350. 6508442

[23] Tagawa N , Takano T , Fukata S , Kuma K , Tada H , Izumi Y , et al. Serum concentration of androstenediol and androstenediol sulfate in patients with hyperthyroidism and hypothyroidism. Endocr J. 2001;48:345‐354. 11523906 10.1507/endocrj.48.345

[24] Ashkar FS , Smoak WM 3rd , Gilson AJ , Miller R . Gynecomastia and mastoplasia in Graves’ disease. Metabolism. 1970;19:946‐951. 5536390 10.1016/0026-0495(70)90041-7

[25] Deepinder F , Braunstein GD . Drug‐induced gynecomastia: an evidence‐based review. Expert Opin Drug Saf. 2012;11:779‐795. 22862307 10.1517/14740338.2012.712109

